# ‘Radical Interpretation’ and the Assessment of Decision-Making Capacity

**DOI:** 10.1111/japp.12035

**Published:** 2013-08-13

**Authors:** Natalie F Banner, George Szmukler

**Affiliations:** Centre for Humanities & Health, King's College LondonUK; Institute of Psychiatry, King's College LondonUK

## Abstract

The assessment of patients' decision-making capacity (DMC) has become an important area of clinical practice, and since it provides the gateway for a consideration of non-consensual treatment, has major ethical implications. Tests of DMC such as under the Mental Capacity Act (2005) for England and Wales aim at supporting autonomy and reducing unwarranted paternalism by being ‘procedural’, focusing on how the person arrived at a treatment decision. In practice, it is difficult, especially in problematic or borderline cases, to avoid a consideration of beliefs and values; that is, of the substantive content of ideas rather than simple ‘cognitive’ or procedural abilities.

However, little attention has been paid to how beliefs and values might be assessed in the clinical context and what kind of ‘objectivity’ is possible. We argue that key aspects of Donald Davidson's ideas of ‘Radical Interpretation’ and the ‘Principle of Charity’ provide useful guidance as to how clinicians might approach the question of whether an apparent disturbance in a person's thinking about beliefs or values undermines their DMC. A case example is provided, and a number of implications for clinical practice are discussed.

## Introduction

The assessment of a patient's ability to make a decision regarding his or her treatment has become an important area of clinical practice with major ethical implications. The Mental Capacity Act (2005)^1^ for England and Wales sets out a test of capacity that intends a balance to be struck between respecting patient autonomy and fulfilling a duty of care. Capacity (or guardianship) legislation of this kind allows clinicians to intervene if patients are unable to make decisions for themselves (even against their expressed wishes), with a further requirement that decisions made on their behalf must be judged to be in their ‘best interests’. The MCA is considered to be a successful model of capacity legislation, gaining recognition and influence across many jurisdictions.^2^ Similar to internationally used assessment aids such as the MacCAT-T,^3^ it sets out a test of capacity based on procedural criteria that aim to track the process a person uses to come to a decision, irrespective of its content. Since the implementation of the MCA, clinical, legal and philosophical interest in capacity has gained momentum, presenting challenges that are likely to affect everyday clinical practice. Ascertaining whether or not a person is capable of making a particular decision can be especially difficult in psychiatry, where a mental disorder may undermine decision-making in varied ways. There are good reasons for thinking that capacity assessments cannot be reduced to measures of cognitive functioning and value-free judgements,^4^^,^^5^ but at the same time clinicians must avoid undue paternalism in evaluating a person's beliefs, values and decisions.

In this article, we argue that in difficult and borderline cases, capacity assessment is complex and ultimately based on a judgement, one involving *interpretation*, that cannot be reduced to a set of procedural, tick-box criteria. Drawing on the philosopher Donald Davidson's theoretical framework for the conditions of possibility on understanding the words and actions of others, we consider parallels that may illuminate clinical judgements about capacity; these are intended to provide prompts for critical reflection on clinical practice, rather than the basis for an alternative model of capacity assessment. These insights are illustrated using a case study to question what standard of objectivity it is possible to achieve in assessing decision-making capacity (DMC); and they may point to the kinds of information and understanding clinicians ought to seek when assessing their patients' capacity. The criteria for tests of capacity such as the MacCAT-T and the test in the MCA may generate the illusion of objectivity, but we suggest that the complexities of clinical judgement ought to be acknowledged and embraced if such assessments are to track when and how capacity might be impaired.

Finally, we examine some clinical implications following on from our analysis of the assessment of decision-making capacity — what kind of training might be required, checks on unacceptable paternalism, and the time that might be required.

## Capacity Assessment

Whilst decision-making capacity must be presumed until proven otherwise, if a patient fails to understand and retain information relevant to the decision, ‘use or weigh’ (on the MCA) that information in the process of coming to a decision, or communicate that decision, a judgement of incapacity can be made. On the MacCAT-T, ‘appreciation’ and the ability to ‘reason’ with the information occupy similar roles to ‘use and weigh’ in the MCA. These criteria have a well-established pedigree in many tests of capacity.^6^ Assessment should be based on evaluating the process a patient uses to arrive at a particular decision, rather than the content of the decision itself.^7^ In this respect, it is intended to be ‘normatively-neutral’; the test does not prescribe the decisions that ought to be made in order for a patient to be deemed to have capacity, although in the development of the MCA, the Law Commission struggled with how to develop this non-normative model of capacity.^8^ Such a procedural test is a laudable aim, as it prevents clinicians from deeming a person to lack capacity because they make a seemingly unwise decision or refuse treatment that is medically recommended. If the decision-making process is intact, the patient is entitled to freely decide.

There are, nonetheless, reasons for doubting that it is either possible or desirable to assess DMC on purely procedural and non-normative grounds. Particularly (but not uniquely) in psychiatry, a patient's decision may be based on unusual or bizarre beliefs or values, but procedural criteria cannot necessarily distinguish whether these impair or undermine the process of decision-making.^9^ For example, if a patient holds paranoid delusional beliefs about the purposes of a proposed treatment and thus refuses a potentially life-saving medical intervention, decision-making capacity may be called into question. However, it could be argued either way whether the patient's decision-making process is sound according to capacity test criteria. On the one hand, his decision does logically follow from his beliefs; it makes sense that he refuses a medical treatment if he does not trust his doctors and believes the treatment will intentionally harm him. On this basis his cognitive function (relevant to a judgement about his capacity) is intact, and he could be said to understand, use, weigh, appreciate or reason with the relevant information in coming to a decision. It does not matter for the assessment of capacity whether the decision is one that would be considered wise or sensible, or medically sound. On the other hand, it could be argued that a failure to appreciate the falsity of his delusional beliefs and his rejection of the likely harms that may befall him should he refuse the treatment indicate the patient's capacity may be undermined. The problem for the capacity test is that clinicians could argue either way, depending on how the requirements are understood. The resulting judgements can be controversial and ethically challenging as they are right at the heart of the balance between a clinician's duty of care and the rights of patients to act autonomously.^10^ This difficulty may be especially evident in cases involving depression, anorexia nervosa and some psychotic illnesses, where capacity judgements may be complex, carry high stakes, and be contingent upon evaluative judgements about what the patients believes, values and wishes to do.^11^

It is apparent that even if the legal test for capacity is procedural in nature, clinical decision-making, and judgements by the courts, in fact do take into account the substantive features of a patient's decision-making when judging capacity.^12^ Indeed, the developers of the MacCAT-T state that if a decision-making process is influenced by a ‘gross distortion of reality’,^13^ capacity may be impaired, thus acknowledging that substantive judgements can legitimately be made about a person's beliefs and values when capacity is assessed. Case law provides a rich seam of examples to illustrate the borderline where particular beliefs may be judged to impair a person's capacity. In a judgement granting a hospital the right to override a patient's refusal for a medically necessary hysterectomy, the court stated: ‘a compulsive disorder or phobia may prevent the patient's decision from being a true one, particularly if conditioned by some obsessional belief or feeling which so distorts the judgment as to render the decision invalid’.^14^

Here, the patient believed that she was childless and refused the treatment on the grounds that she wanted children. The fact that she had two grown up children indicated her capacity to make that decision was impaired. Patients may make decisions that, if understood as following from the beliefs and values that they hold, are entirely reasonable and follow a clear process. It is the beliefs and values themselves that, being the product or result of a disturbance of mental functioning ‘distort’ the decision-making process.

However, incorporating judgements about a patient's beliefs (and values) may risk straying too far into the realms of medical paternalism. It is undesirable to require of patients that they only make epistemic commitments that are in line with their clinicians' or others' beliefs, and indeed case law again indicates that irrationality itself may not necessarily be a bar to capacity, even if a decision is ‘so outrageous in its defiance of logic or of accepted moral standards that no sensible person who had applied his mind to the question to be decided could have arrived at it’.^15^

For many instances of mental disorder, a denial by the patient that they are ill, or that they need the proposed treatment, does not necessarily constitute a rejection of true facts. Psychiatric diagnoses and treatments are frequently controversial, and patients and clinicians may differ significantly in their evaluations; disagreeing with a medical opinion ought not to be taken as evidence of a failure to appreciate normatively significant facts about one's condition. There is an intuitive difference between disbelieving a doctor's opinion because one is detached from reality or impervious to reason owing to a mental impairment and the natural tendency to critically reflect upon and assess the medical expertise one is offered.^16^ But it is not clear how a decision influenced by merely unusual beliefs might be distinguished from one that reflects impaired capacity — how ‘pathology’ can be demarcated from eccentricity or cultural variation.^17^

In many cases, the procedural capacity criteria will be entirely sufficient to make a reliable judgement about a patient's capacity. A finding from a study of capacity in which G. Szmukler was an investigator^18^ showed that 60% of recently admitted psychiatric inpatients who were judged to lack capacity following a detailed research assessment (using the MacCAT-T to inform a clinical judgement) demonstrated a clear lack of understanding about why treatment was being proposed and what it would involve, or were unable to retain information long enough to articulate a decision, or were unable to make a choice. However, in some cases, where cognitive functioning appears to be intact, assessments of DMC face a substantial challenge that is underpinned by a conceptual difficulty. The procedural criteria on which tests of capacity are based appear to underdetermine the way in which decision-making processes ought to be assessed, which could result in opposite judgments being made depending on how the content of patient beliefs, values and decisions are considered. There is no ‘gold standard’ for assessing capacity, and diagnostic tools designed to aid its assessment acknowledge the importance of clinical judgement.^19^ Yet there is little guidance as to how clinicians may negotiate this difficult area.

Against the backdrop of the clinical dilemma, and taking into account that capacity assessment revolves around what a person believes, understands and wants in coming to a decision, we pose the following question: when assessing capacity, how might evaluative judgements about the quality of a person's beliefs, values, desires^20^^,^^21^ and decisions be understood, without straying down the path of unwarranted paternalism, and what degree of objectivity in this judgement is it possible to achieve?

## Donald Davidson and Interpretation

To critically approach this question we draw on some ideas from Donald Davidson's theory of interpretation, originally conceived as a project in the philosophy of mind and language.^22^ ‘Interpretation’ here is a shorthand for the process of seeking to make sense of people's utterances and intentional actions from a third-person perspective using the language and concepts of beliefs, values, decisions and actions, that does not depend on a prior grasp of substantial theory or knowledge. Interpretation can be understood as part of the process of forming judgements about a person's reasons for an action or decision, enabling an interpreter to ascertain whether there is a process occurring that renders that action or decision intelligible. Although Davidson's project is a theoretical abstraction of the conditions of possibility for interpretation in a radical scenario, its insights have previously been taken to be directly applicable to quotidian circumstances of seeking to understand one another, supplying an epistemological framework through which to build interpretation.^23^^,^^24^

The interest in this article is in instances where it is by no means clear if a person's decision-making process is intact or whether it is impaired as a result of a disruption or disturbance caused by a putative mental disorder. In exploring the process of interpretation the aim is thus to scrutinise the boundaries of intelligibility and sense-making that are breached when communication and understanding break down. This is in order to ascertain whether or not there are necessary conditions underpinning the possibility of interpreting another intentional agent. If such conditions exist, they could supply normative guidance for understanding a person's decision-making processes even if the beliefs and values that person holds look very different to one's own. We suggest that Davidson's arguments can thus provide a route into examining whether any objective standards might be operating implicitly when a decision-making process looks as though it might have gone awry, and certain beliefs or ways of thinking are the result of a mental disturbance that compromises a patient's capacity. The aim of seeking to apply Davidson's insights to clinical practice is to shed light on the normative standards disciplining how we perceive the intelligibility of another person's decisions and actions. The analysis here is not intended to provide an alternative or competing method of assessing capacity. Rather its aim is to complement and enrich standard tests of capacity, in particular providing substance to what it might mean to fulfil or fail the criterion of using, weighing or appreciating information.

Davidson generated an idealised method for understanding the utterances of a radically unfamiliar speaker without any prior knowledge of the speaker's language or his mental states. His method of ‘Radical Interpretation’ makes a claim for what assumptions an observer would necessarily have to make in order to begin the task of interpreting such a person's beliefs, utterances and actions. It aims to shed light on the role that beliefs ordinarily play in behaviour (including actions, utterances and decisions) and how they are related to one another, to other psychological states, and importantly, to the world.

In this radical scenario, the interpreter is licensed to make certain assumptions about the speaker's beliefs and language, owing to the fact that the speaker and interpreter share features of the perceptual environment, and certain psychological features, in virtue of being agents capable of speaking and acting. There are intrinsic, normatively governed relationships between the meanings of the speaker's utterances, the beliefs he holds and the actions he performs, on the basis of which the interpreter can frame his attempts to understand the speaker's behaviour: ‘interpretation depends on reading some of the norms of the interpreter into the actions and speech of those he interprets’.^25^ Thus interpretation is an explicitly normative project, aimed at making sense of a person's behaviours by reference to the kinds of thing he ought say and think under specific circumstances. This sense of ‘ought’ is not, however, strongly prescriptive, being disciplined by the normative standards of language and behaviour the interpreter himself is subject to, which may allow for a wide range of plausible interpretations. Davidson's construal of what these norms are is known as the Principle of Charity.

Charity consists of two distinguishable constraints on interpretation: ‘we must assume that a speaker is by and large *consistent* and *correct* in his beliefs’.^26^ Broadly, these conditions of consistency and correctness are termed ‘*Coherence*’ and ‘*Correspondence*’ respectively.^27^ In other words, a person's beliefs, intentions and decisions to act broadly hang together as a more or less coherent whole, and they largely reflect what is true. An interpreter is thus guided by these norms as a first step in attempting to understand what meaning can be attributed a speaker's actions and utterances. It is important to note these caveats of ‘more or less’ and ‘largely’; they reflect the fact that interpretations of individual instances of behaviour are imprecise, may always be subject to revision, and need not be *strictly* bounded by demands for truth and coherence.

One significant consequence of Davidson's view of the relation between beliefs, meanings and the world is that the mental realm is holistically constituted; that is, elements or items within that realm are individuated only through the relations they bear to other items within that system. This requires an interpreter to take into account and attribute a whole host of related beliefs, utterances and behaviours when attempting to interpret a particular instance of behaviour, because the attribution of a single belief rests on the supposition of many more.^28^ This holism extends throughout an agent's psychology and into his interactions with the physical and social world. It is important not to underestimate the wide horizon over which this holism operates, as it undermines the idea that beliefs can be understood and attributed in isolation from one another, an idea reflected in an influential view of the later Wittgenstein: ‘when we first begin to believe anything, what we believe is not a single proposition, it is a whole system of propositions. (Light dawns gradually over the whole.)’^29^

The injunction to attribute ‘coherence’ and ‘correspondence’ to beliefs does not constrain interpreters only to attribute true beliefs that are perfectly consistent with one another. Our mentality is inherently messy; we are epistemically limited, form beliefs on partial evidence and that may conflict, experience cognitive dissonance, act against our own avowed interests, and make decisions based on emotional rather than rational thought processes. Far from denouncing human agency as imperfect and irrational, the holism implied in Davidson's theory of interpretation allows significant scope for errors of judgement, false beliefs and beliefs that do not cohere well with one another, in the totality of our mental economy. What the standards of Charity do imply is that there must necessarily be a broad background of true and coherent beliefs in order for a speaker to be interpretable at all. Indeed, it is only by taking account of this background of largely true and coherent beliefs, shared by an interpreter, that incoherence, errors or irrationalities could be thrown into relief and recognised.^30^

## Applying ‘Radical Interpretation’ to Capacity

Davidson's account is an explicitly theoretical reconstruction of the grounds for interpretation. However, there are two major insights from this methodology of interpretation that we wish to draw out and seek to apply in the clinical context. One concerns the active role played by the observer in attributing belief and meaning to the speaker. Interpretation is not a matter of an observer discovering objective facts about the speaker's beliefs, values and so forth. Rather, the process of interpretation involves a dialogical encounter, a negotiation to achieve mutual understanding within a shared perceptual environment. The logical and epistemic resources of the interpreter are essential to the process of getting interpretation off the ground; what the interpreter himself believes will necessarily influence how he interprets another. It would be a mistake to attempt to strip these resources away in the interests of achieving objectivity or perspective-neutrality in one's judgement. For Davidson,^31^ this is a transcendental claim about the nature of belief, meaning and the conditions of possibility on interpreting another's behaviour and utterances.^32^

For our purposes, it is the epistemological implications of the claim and the potential application they have for clinical practice that are of interest here. It suggests the clinician, in the context of attempting to understand a patient's beliefs, values and decisions for the purposes of assessing capacity, cannot make an entirely objective judgement that is not predicated upon his own perspective, as an intentional agent capable of behaviours such as believing, wanting, deciding and doing. Clinical interviews take the form of conversations, a back-and-forth between patient and clinician as the latter attempts to gain a fuller insight into the world of the patient and what he believes and wants, in order to ascertain whether he has the capacity to make a particular choice. To treat this process like a simple or reductionist test of cognitive functioning would be misguided, as it would overlook the clinician's own role in the encounter, creating a veneer of objectivity about the judgement being made.

Recall that our primary question concerned how evaluations could be made about a patient's beliefs, values and choices that do not open up the route to unwarranted paternalism. At first glance, acknowledging that the clinician's perspective plays an inherent role in making these evaluations might look as though it risks opening these floodgates. However, two points can be made in response to this concern. Firstly, it is better to acknowledge and bring out into the open implicit influences on clinical judgement, rather than labouring under the illusion that they are normatively neutral and impartial (and as we have argued above, capacity assessments often do go beyond mere evaluation of cognitive functioning). Secondly, acknowledging that the clinician's own ‘perspective’ guides interpretation does not entail the clinician being the arbiter on what the patient *ought* to believe, want or decide. Davidson's methodology provides an account of what has to be the case in order for an agent's behaviour to be understood as intentional at all; the clinician's ‘perspective’ in this regard is simply that of an intentional agent in a particular perceptual environment. The norms guiding interpretation are not strongly prescriptive but are rather the general constraints of attributing broad coherence and correspondence in the patient's overall psychology.

The second, related insight is that the necessary starting assumptions made by the ‘Radical Interpreter’ also reveal something important about the structure of beliefs, potentially offering clinicians a better understanding of how to go about examining the beliefs influencing a patient's decision. In a clinical psychiatric setting, patients may express beliefs or make assertions that are at odds with what they have previously claimed, or that strike an observer as out of character, bizarre, as emerging spontaneously, or held for no apparent reason. Rather than focus on the *content* of belief as informative for identifying a mental impairment, Davidson's insights into the logic and normative structure of beliefs may be taken as counselling us to examine the relations they bear to the patient's other beliefs, utterances, psychological states, and actions, to ascertain whether they occupy the right kind of role in a person's mental economy to form part of a legitimate decision-making process. Exploring the context in which such beliefs arise and their history, and their connectedness with other beliefs, values and behaviours will identify whether they broadly cohere and are capable of supporting, rather than undermining, decision-making capacity.

The epistemic standards of ‘coherence’ and ‘correspondence’ thus provide a framework within which decisions and behaviour, whether unusual or not, can be interpreted and understood. It is only in virtue of the implicit background structure of interconnected beliefs, actions, and so forth, that individual beliefs (or values) can be picked out as normatively inappropriate, and therefore potentially indicative of an impairment that could undermine capacity: a note of discord in an otherwise fairly coherent and harmonious symphony of intentional behaviour.

The conceptual resources are thus available to recognise blips in this psychological landscape in a far more refined manner than simply identifying bizarre content and attempting to grasp whether or not it is indicative of pathology. For instance, the steadfast belief of a female patient with anorexia nervosa that she is fat will likely be intrinsically bound up with numerous other beliefs about how she appears, issues of self-esteem and self-worth, positive aspects of self-control, perceptions of aesthetic ideals, and so on. All of these factors and many more will modulate this belief and each subtly influence what it means and how it impacts upon decision-making. There may be good reasons for adherence to this belief in the face of counterevidence or argument, and it is necessary to seek such possible reasons before determining whether it indicates a capacity-undermining impairment. Far from being so broad and general as to paralyse normative judgements about individual instances of decision-making, the global structure of beliefs provides the backdrop against which potentially capacity-undermining divergences can be identified and explored.

## An Illustration: The Case of Mr Blythe

The following vignette is a composite case, based on clinical encounters with several like patients. We employ it here to illustrate the potential role of a Davidsonian approach to interpretation for understanding a patient's reasoning and decision-making.

Mr Blythe, a 52-year-old financial advisor with a major city firm, was brought to the A&E department by the police under an order of the Mental Health Act. Neighbours had called the police as a result of the disturbance he had created outside his house. From A&E he was admitted under a compulsory order to a psychiatric ward.

### Presenting Problem

According to his wife, for three months Mr Blythe had been behaving unusually. He was generally irritable, but often cheerful, and at times even ‘euphoric’. Although normally a relatively cautious man, recently he had been overspending. For example, he flew to New York for two days to see his favourite ballet dancer perform. While there he stayed in an expensive hotel, and invited a number of people, whom he met for the first time at the theatre, to dinner. On his return, Mr Blythe had placed a deposit on a new Jaguar, even though he already had a well-appointed car provided by his firm. Mrs Blythe had been told by one of the firm's partners that Mr Blythe had been ringing clients in the early hours of the morning with ‘exciting’ investment suggestions. Mrs Blythe had become increasingly annoyed by her husband's irritability, unwise decisions and his refusal to see a doctor, and finally locked him out of the house after an angry argument. It was Mr Blythe's shouting following this incident that led to the neighbours calling the police. Mr Blythe's two daughters, one of whom was still living at home, had stopped talking to him some weeks previously. Mr Blythe's wife said she had decided she would not longer live with him.

### Mental State Examination

On admission, Mr Blythe was generally polite and friendly, but quickly became irritable if a request was not immediately granted. He appeared mildly elated and said he felt ‘great’. While his speech was pressured at times, it was coherent. Some of his responses to questions about recent events and the need for treatment were as follows:

Clinician: Why did your wife lock you out?

Mr Blythe: *It was just an argument. She can be unreasonable. She's never really understood me. But, don't worry, we'll make it up. She'll see I was right.*

Clinician: Why were the police called?

Mr Blythe: *Nosey neighbours; everyone dislikes them.*

Clinician: Do you think you have been overspending?

Mr Blythe: *Why not? I'm a generous guy. I work hard, and I'm on a good salary. I'm very talented at spotting good investments. Soon my firm will offer me a partnership.*

Clinician: Was your trip to New York wise?

Mr Blythe: *She's a great dancer, beautiful to watch. I missed her at Covent Garden.*

Clinician: Did you think you might have a relationship with her?

Mr Blythe: *No, it's not really possible. But if she knew me she might very well fall in love with me.*

Clinician: Do you think ringing clients at 1 am was a good idea?

Mr Blythe: *Would you mind being called if you were going to make a million?*

Clinician: Have you made much money recently?

Mr Blythe: *Not really yet, but the big investments haven't born fruit yet. I'm certain they will. I can pick the winners; I've got a real nose for it. It's more than just analysis; it's a special talent, maybe even God-given.*

Clinician: Is it possible that you stand to lose a lot, perhaps everything?

Mr Blythe: *Yes, of course it's possible. But when you've got a huge chance of winning, it's worth taking the risk.*

Clinician: Family relationships have broken down?

Mr Blythe: *They'll come round when they see how successful I've been. It's a do or die world out there; they don't really understand it.*

Clinician: Do you need treatment?

Mr Blythe: *That's a ridiculous idea. There's nothing wrong with me. Are you trying to ruin my future? I know I'm going to make money. I'll probably have my own firm soon. Being in hospital could spell the end. I'll lose my job, my clients. I'll be finished. I don't think you understand what is at stake here.*

## Analysis of Mr Blythe's Capacity

The key question facing clinicians in this scenario is whether Mr Blythe has the capacity to make the decision to refuse admission to hospital and treatment. Is his refusal a legitimate one based on an understandable desire to avoid hospitalization and a reasonable belief that he does not require treatment? Or is his reasoning and decision-making indicative of a capacity-undermining mental disturbance, so that involuntary admission and treatment could be recommended if it were decided it was in his ‘best interests’?^33^

This case represents a common clinical dilemma. We do not seek to answer the question of Mr Blythe's capacity definitively, but rather to draw on Davidson's insights into the nature of interpretation to point us in the direction of identifying the kinds of questions a clinician ought to ask, and the depth of background understanding required to ascertain, with a reasonable degree of justification, whether Mr Blythe possesses or lacks capacity. It is our contention that rather than narrowing the scope of the assessment down to the specific issue at hand and the contents of the beliefs and values entering into the decision process, Davidson's arguments about the holistic context for interpretation direct us towards expanding the horizon of enquiry, to gain a broader understanding of how the factors impinging upon a person's decision process may (or may not) fit into the decision-making context.

On a narrow, procedural reading of the criteria for capacity, Mr Blythe does understand some of the consequences of being detained and treated: he agrees that his actions carry serious risks, but hospitalisation would, as he correctly points out, likely impact severely on his career and earning potential. He is capable of responding to the clinician's questions and his answers to questions regarding his work and family life follow clearly. Mr Blythe works in an industry characterised by values that reward self-confidence, risk-taking and a degree of bullishness in one's convictions. In this context, extravagant spending, risk-taking, dismissing marital concerns and calling clients with investment ideas can be seen to be reasonable and even ordinary, and he is convinced his actions will be justified by his successes. We might interpret Mr Blythe as attempting to impress at work and thus achieve a promotion through prioritising work over his family temporarily, and perhaps treating himself to personal indulgences as a reward or to impress others with the accoutrements of success. Mr Blythe's predicament could be seen as a complex but by no means unusual set of circumstances, with competing pressures on his time and energies. Given this context presented by the brief case history, what further analysis might be needed in order to assess his capacity as objectively as possible?

Radical Interpretation counsels us to examine the relations between a person's utterances and his further beliefs and behaviour, as it is only through understanding these connections that inconsistencies that flag up a possible impairment may be identified. Individually, each of Mr Blythe's beliefs, behaviours and responses to questions could be taken as reasonable and understandable; but we argue that expanding the horizon of enquiry provides a fuller picture of his recent behaviour, and furthermore one that is required in ascertaining his capacity to decide about treatment.

Reflecting on the behavioural changes that have brought him to the attention of mental health services, the nature of his epistemic and evaluative commitments have evidently shifted significantly in the past three months, and we focus our analysis here on one particular aspect of his behaviour that possibly signals a discord in his commitments: the level of conviction in his business acumen, reflected in his behaviour in phoning clients in the early hours and his apparent bullish certainty about his investments. The conviction with which he relays his investment beliefs identifies an area of his thinking that warrants closer scrutiny, perhaps interpretable as grandiosity and a disturbance that might undermine capacity. This is because the cluster of behaviours surrounding these beliefs appears at first sight to be relatively isolated from considerations of the consequences that could follow from them. For example, Mr Blythe does not seem to have considered that it is possible that phoning clients in the middle of the night would annoy them and lead to doubts about his professionalism, rather than encouraging investment. This would defeat his intentions.

Further discussion with Mr Blythe showed that his thinking did not appear to accommodate this possibility. Normatively, there is something amiss here in the fact that Mr Blythe was unwilling or unable to perceive the logical inconsistency between his intentions and his actions. He did not think through the obvious consequences of his investments failing, or of adverse effects on his clients' attitudes towards him. This was despite serious risk-taking in which the consequences of failure, as confirmed during interviews with his wife and a senior partner in his firm, could be catastrophic for him. His colleague said that Mr Blythe's chances of becoming a partner in the firm were anyway slim. Mr Blythe could not offer any reasons for believing that despite the, at best, modest record of investment successes described by his colleague, he should now be outstandingly successful. He just ‘knew’ it. Mr Blythe had no contingency plans in mind, even when encouraged to explore them, to deal with the possibility of serious losses, including the loss of his job. Further, Mr Blythe had always been a dedicated worker, but prioritising work to the extent of alienating his wife and daughters, possibly permanently, was a marked change in values, again with potentially serious consequences. Mr Blythe would not, or could not, discuss what these consequences would mean for him. The Blythes had been married for over 20 years and their relationship had been reasonably stable. Mr Blythe, when asked about his social life and support network, stated that his wife was his only friend.

Thus his apparent ability to think about admission and treatment was called into question by his broad lack of appreciation of the consequences of his risk-taking behaviour for him.

The suggestion that this contextual information should be brought to bear on an assessment of capacity may appear to provide a recipe for rampant paternalism: for clinicians to flag up any inconsistencies in a patient's thinking or disagreements with the clinician, even beyond the decision at hand, as evidence of incapacity. This would be an extremely undesirable outcome and would set the bar for capacity too high for most of us to reach. However, we suggest that awareness of these contextual features of Mr Blythe's background and recent history in fact illuminate the decision process at hand, and in effect increase the scope available to clinicians to interpret and understand a patient's thinking as charitably as possible. The risk of paternalism may indeed be reduced if clinicians are counselled to give serious consideration to beliefs that initially seem bizarre or unwarranted and indicative of a capacity-undermining impairment. It may often be the case that beliefs or values that one might write off as symptoms of a psychiatric disorder are legitimately and coherently held by the patient: without exploring the patient's related beliefs, values and choices, it would be impossible to make this distinction. In the case under consideration, contextual features indicate that Mr Blythe significantly lacks an appreciation of his current predicament and has a difficulty in thinking about his future self, which may point to a capacity-undermining impairment.^34^ There is evidence, in Davidson's terms, of difficulties with both ‘coherence’ (his beliefs and behaviour are out of the range of his past beliefs and behaviour, and are not connected by beliefs or values that, despite probing, could reasonably account for them) and ‘correspondence’ (his belief in the success of his investments is not apparently based on anything like a realistic appraisal of his situation). Mr Blythe's beliefs about the need for treatment are significantly related to these difficulties and, on the basis of the account we have outlined, potentially point to a lack of capacity in this regard.

We are careful to avoid the implication that the Davidsonian notions of ‘coherence’ and ‘correspondence’ should be taken as prescriptive principles for judgements of capacity. This would precisely defeat the objective of encouraging critical reflection and an awareness of the importance of context in making such judgements. Rather, we suggest that these norms be used to provide guidance in broadening out a clinician's scope of enquiry, to identify not just the content of seemingly bizarre or logically incoherent thoughts but also the relations they bear to other beliefs and values the patient holds, and the decisions he makes. Perfect coherence and belief only in true facts are not the standard by which we judge the decisions of others, but where DMC has been brought into question, exploring the coherence between beliefs or correspondence with the shared perceptual world may help the clinician to ascertain whether or not a patient is using, weighing or appreciating information in coming to a decision.

## Discussion

We have suggested that in difficult cases, judgements about patients' decision-making processes rely on an evaluation of the patient's relevant beliefs, values and the choices made. However, taking substantive content into account need not lead to unwarranted paternalism, as the way in which we judge these psychological attitudes is not arbitrary but normatively governed. Drawing on parallels with Davidson's method of interpretation can provide insights into the normative, relational structure of a patient's beliefs, values and decisions. We cannot generate a context-independent checklist for determining capacity, but this does not imply that the judgement is arbitrary: there is content to the idea that there is normative shape to the appreciation of a person's decisions. Taking the broader holistic context into account, we can gesture towards the kinds of questions a clinician conducting an assessment of capacity ought to bear in mind. For example, one might query the consistency of the patient's decision-influencing beliefs with his broader worldview, the empirical sensibility such beliefs have and whether they are amenable to revision or argument, and the cultural acceptability and rationality of the values impinging upon decision-making, to name but a few possible considerations.

It is only if we acknowledge the context surrounding each decision and the relational nature of the beliefs and values impacting on decisions that we are in a position to identify where anomalies occur that might indicate impairment to decision-making capacity. Whilst we cannot provide a clear-cut prescriptive specification of how capacity judgements ought to work, there are nonetheless grounds for seeking a wider scope of enquiry in conducting a capacity assessment than the cognitive conception of capacity alone might permit. The assessment we have discussed is most pertinent to the ability to ‘use or weigh’ component of capacity in the Mental Capacity Act 2005; or to ‘appreciation’ and ‘reasoning’ in the account given by the influential MacCAT-T test of capacity.

Although Mr Blythe might have received a diagnosis of ‘hypomania’, we have not placed much weight on this consideration. Determinations of capacity involve a ‘functional’ assessment and are not based on ‘status’, such as being diagnosed with a particular disorder. A diagnosis may point to possible kinds of disruption of thinking that might be explored, but offers no more than that and ought not to influence the assessment of capacity.

Davidson recognised an inherent ‘indeterminacy’ in interpretation. Perhaps an utterance that thus far appears not understandable, may not remain so with further probing of the person's beliefs and values. This is certainly a significant consideration, but in the real-world situation of a clinical encounter, an important decision may need to be made whether a person lacks capacity to make a treatment decision, with the consequence, if other conditions are met, that non-consensual treatment may be justified. The aim is to reach a point in the dialogue where it is reasonable to conclude that there is a significant-enough breach in the relational structure of the patient's beliefs and values. A degree of objectivity appropriate for this purpose is the goal, and this can be tested by asking others (for example, colleagues, those who know the patient, a court) whether the evidence is sufficiently convincing. Freyenhagen and O'Shea^35^ suggest the possibility of including a process of institutional oversight involving among others, those with mental disorders and people close to them. They propose the principle that those affected should have a voice in deciding how evaluations should proceed and be interpreted. Thus the triangulation of judgement between a clinician and others may be the most appropriate method of achieving a reliable and valid judgement that protects against unacceptable paternalism, rather than attempting to minimise clinical judgement through convergence on rating scales of cognitive elements of capacity.

Psychiatrists may recognise that this approach shares some features with Karl Jaspers' distinction between ‘understanding’ and ‘explanation’.^36^ ‘Understanding’ (*verstehen*), or grasping meaningful connections, asks how ‘one psychic event emerges from another’, and is understood by another directly, irreducibly, by empathy — that is by imagining oneself in the place of the other. However, understanding sometimes reaches an end-point where despite every effort to empathise with the subject's predicament — taking account of his history, personality and so on — his experiences or behaviour are *un-understandable* — ‘one psychic event follows another quite incomprehensibly; it seems to follow arbitrarily rather than emerge’. Thus we encounter signs of a mental illness, and to go further we must resort to ‘causal explanation’ (*erklären*) — to see it from ‘the outside’, following different principles, namely the methods of the natural sciences, seeking to discern regularities between phenomena which are objective, law-like, and general. Thus, a biological explanation, for example, may be sought. Hence the distinction drawn by Jaspers between un-understandable illness ‘process’ and understandable ‘reaction’, the former representing a discontinuity or breach in the subject's psychic life. In the terms we are considering in this article, the influence of such discontinuities on treatment decision-making may result in impaired capacity.

If the assessment of capacity, especially in difficult cases, involves more than a procedural test of cognitive functioning or a reductionist ‘tick-box’ approach, there are some important practical implications. First, understanding the principles of the approach we have outlined, although based on shared norms of interpretation, probably requires training that goes beyond simple interview skills. Clinicians need the skills for this particular kind of conversation as they are frequently charged with making capacity assessments. Second, capacity assessment involves an interaction between the assessor and the person being assessed. Assessors need to be alert to the fact that the process of exploration of a person's system of beliefs and values may challenge some of them, and possibly result in their being changed or revised.^37^ They are not necessarily immutable characteristics of the person that require only to be uncovered. Furthermore, understanding the interactional nature of the discussion about treatment is in tune with the idea of ‘supported decision-making’ as expressed in the UN Convention on the Rights of Persons with Disabilities.^38^ Clarifying with the patient the beliefs and values relevant to a decision may be important in helping the person to decide with greater assurance, one way or the other: this is the standard of objectivity one can hope to achieve in assessment, though it is not guaranteed or legitimated by reference to an index of cognitive functioning.

Finally, it will be obvious from our analysis that a capacity assessment may require substantial time. Understanding a person's individual and complex system of epistemic and evaluative commitments may require more than a single interview. Observing the actions of the person, as well as consulting with others who know them well may be important. Such procedures do not fit easily with time constraints imposed by A&E departments. Nor do they comport well with trends in clinical practice to devise straightforward algorithms, checklists or ‘decision-aids’ to assist, standardise and speed-up clinical decision-making. Nonetheless, for decisions such as these, which potentially involve treating a person against their wishes, assessment ought to be as thorough as possible. It is our contention that good practice can only proceed by taking seriously the holistic, contextual nature of a person's decision-making process.

## References

[1] Mental Capacity Act (2005). http://www.legislation.gov.uk/ukpga/2005/9/pdfs/ukpga_20050009_en.pdf.

[2] Victorian Law Reform Commission (2012). Guardianship: Final Report Law Reform Commission, Melbourne. http://www.lawreform.vic.gov.au/projects/guardianship-final-report.

[3] Grisso T, Appelbaum PS, Hill-Fotouhi C (1997). ‘The MacCAT-T: A clinical tool to assess patients' capacities to make treatment decisions’. Psychiatric Services.

[4] Banner NF (2012). ‘Unreasonable reasons: Normative judgements in the assessment of mental capacity’. Journal of Evaluation in Clinical Practice.

[5] Freyenhagen F, O'Shea T (2013). ‘Hidden substance: Mental disorder as a challenge to normatively neutral accounts of autonomy’. International Journal of Law in Context.

[6] Cairns R, Maddock C, Buchanan A, David AS, Hayward P, Richardson G, Szmukler GS, Hotopf M, Appelbaum PS, Grisso T (1995). ‘Reliability of mental capacity assessments in psychiatric in-patients’ ‘The MacArthur Treatment Competence Study. I: Mental illness and competence to consent to treatment’. British Journal of Psychiatry Law & Human Behavior.

[7] Department of Constitutional Affairs (2007). Mental Capacity Act 2005: Code of Practice.

[8] Law Commission [England and Wales] (1991). *Consultation Paper No. 119*: *Mentally Incapacitated Adults and Decision-Making: An Overview*.

[9] Tan J, Stewart A, Fitzpatrick R, Hope T, Owen GS, Cutting J, David AS (2006). ‘Competence to make treatment decisions in anorexia nervosa: Thinking processes and values’ ‘Are people with schizophrenia more logical than healthy volunteers?’. Philosophy, Psychiatry, Psychology British Journal of Psychiatry.

[10] David AS, Hotopf M, Moran P, Owen G, Szmukler G, Richardson G (2010). ‘Mentally disordered or lacking capacity? Lessons for management of serious deliberate self harm’. British Medical Journal.

[11] Owen GS, Freyenhagen F, Richardson G, Hotopf M (2009). ‘Mental capacity and decisional autonomy: An interdisciplinary challenge’. Inquiry.

[12] Banner NF (2013). ‘Can procedural and substantive elements of decision-making be reconciled in assessments of mental capacity?’. International Journal of Law in Context.

[13] Grisso T, Appelbaum P (2006). ‘Appreciating anorexia: Decisional capacity and the role of values’. Philosophy, Psychiatry & Psychology.

[14] (2006). Trust A and Trust B v H (An Adult Patient).

[15] (1997). Re MB (Medical Treatment).

[16] Grubb A, Grubb A, Laing J (2004). ‘Consent to treatment: Competent patient’. Principles of Medical Law.

[17] Holroyd J, Radoilska L (2012). ‘Clarifying capacity: Values and reasons’. Autonomy and Mental Disorder.

[18] British Journal of Psychiatry.

[19] Kim SYH (2006). ‘When does decisional impairment become decisional incompetence? Ethical and methodological issues in capacity research in schizophrenia’. Schizophrenia Bulletin.

[20] Davidson is inconsistent in his use of the terms ‘desire’ and ‘value’, (or pro-attitudes), often appearing to use them interchangeably. For the purposes of this analysis, both desires and values are subject to the constraints of interpretation, and interpretable in the same way as beliefs, although this is of course a contentious claim.

[21] Lillehammer H (2007). ‘Davidson on value and objectivity’. Dialectica.

[22] Davidson D (2001). Radical interpretation ’ (1973) in Inquiries into Truth and Interpretation.

[23] Ludwig K, AR Mele P (2004). Rationality, Language and the Principle of Charity ’ in The Oxford Handbook of Rationality.

[24] Henderson DK (1991). ‘Rationalizing explanation, normative principles, and descriptive generalizations’. Behavior & Philosophy.

[25] Davidson D (1994). ‘Radical interpretation interpreted’. Philosophical Perspectives, 8, Logic & Language.

[26] Davidson D (1973). Psychology as philosophy Essays on Actions and Events.

[27] Davidson D, Davidson D, Davidson D, Davidson D, Davidson D (2001). A coherence theory of truth and knowledge (1983) in Subjective, Intersubjective, Objective ‘Three varieties of knowledge’ ‘Mental events’ ‘True to the facts’ ‘Truth and Meaning’.

[28] Davidson D (2004). Paradoxes of irrationality ’ (1982), in Problems of Rationality.

[29] Wittgenstein L, Anscombe GEM, Wright von GH (1969). On Certainty.

[30] Davidson D (1985). ‘Incoherence and irrationality’ *Problems*.

[31] Davidson D, Child W (1973). ‘Psychology as Philosophy’ *Essays* Anomalism, rationality, and psychophysical relations in his Causality, Interpretation & the Mind.

[32] Davidson pitches this claim at the levels of both metaphysics of intentionality and the epistemology of interpretation. However, we do not require this more controversial aspect of his claim in order to make the epistemological point that an interpreter's own beliefs and values will necessarily be recruited as he attempts an interpretation of another's utterances and behaviour.

[33] ‘Best interests’ is a controversial subject that we cannot deal with in this article. The interpretation that we favour is that if the patient lacked DMC, non-consensual treatment would be justified if the patient would have chosen that treatment in this predicament if capacity had been retained. An ‘advance directive’ would, for example, offer good evidence of such a choice.

[34] Craigie J, Coram A, Vincent N (2012). Irrationality, mental capacities and neuroscience. Neuroscience and Legal Responsibility.

[35] Freyenhagen F, O'Shea T (2013). ‘Hidden substance: Mental disorder as a challenge to normatively neutral accounts of autonomy’. International Journal of Law in Context.

[36] Jaspers K (1959). General Psychopathology. German edn., trans. J. Hoenig & M. W. Hamilton.

[37] Szmukler GS (2009). “Personality disorder” and capacity to make treatment decisions’. Journal of Medical Ethics.

[38] United Nations Convention on the Rights of Persons with Disabilities (2006). http://www.un.org/disabilities/convention/conventionfull.shtml.

